# The interaction of *Akkermansia muciniphila* with host-derived substances, bacteria and diets

**DOI:** 10.1007/s00253-021-11362-3

**Published:** 2021-06-14

**Authors:** Tatsuro Hagi, Clara Belzer

**Affiliations:** 1 Institute of Livestock and Grassland Science, National Agriculture and Food Research Organisation (NARO), 2 Ikenodai, Tsukuba, Ibaraki, 305-0901 Japan; 2 Laboratory of Microbiology, Wageningen University and Research, 6708 WE Wageningen, The Netherlands

**Keywords:** *Akkermansia muciniphila*, Bile acid, Diet, Gut bacteria, Host-derived substances, Prebiotic, Medicine

## Abstract

**Abstract:**

Trillions of microbes inhabit the human gut and build extremely complex communities. Gut microbes contribute to host metabolisms for better or worse and are widely studied and associated with health and disease. *Akkermansia muciniphila* is a gut microbiota member, which uses mucin as both carbon and nitrogen sources. Many studies on *A*. *muciniphila* have been conducted since this unique bacterium was first described in 2004. *A*. *muciniphila* can play an important role in our health because of its beneficial effects, such as improving type II diabetes and obesity and anti-inflammation. *A*. *muciniphila* establishes its position as a next-generation probiotic. Besides the effect of *A*. *muciniphila* on host health, a technique for boosting has been investigated. In this review, we show what factors can modulate the abundance of *A*. *muciniphila* focusing on the interaction with host-derived substances, other bacteria and diets. This review also refers to the possibility of the interaction between medicine and *A*. *muciniphila*; this will open up future treatment strategies that can increase *A*. *muciniphila* abundance in the gut.

**Key points:**

*• Host-derived substances such as bile, microRNA and melatonin as well as mucin have beneficial effects on A. muciniphila.*

*• Gut and probiotic bacteria and diet ingredients such as carbohydrates and phytochemicals could boost the abundance of A. muciniphila.*

*• Several medicines could affect the growth of A. muciniphila.*

## Introduction

Beneficial microbes, probiotics such as bifidobacteria and lactic acid bacteria, can sustain or improve host health by producing antimicrobial substances, immunomodulation and competition for adhesion to host cells with pathogens (Servin 2004). Commensal gut bacteria also can sustain host health. Short-chain fatty acids (SCFAs) such as propionate and butyrate, produced by commensal gut bacteria such as *Bacteroidetes*, *Firmicutes*, *Actinobacteria* and *Verrucomicrobia*, are used as a source of energy and immunomodulation in the host (Louis and Flint 2017). In return for these effects or to foster gut bacteria, substances such as mucin, maternal milk and bile, which can all be growth factors for bacteria in the gut, are provided by the host (Aakko et al. 2017; McLoughlin et al. 2016; Wahlström et al. 2016). Mucin is an energy source for mucin-degrading bacteria such as *Bacteroides* and *Akkermansia* (Crouch et al. 2020). Bile acids strongly link host physiology with bacterial metabolism. Primary bile acids are secreted to the gut by the liver for the absorption of lipid from diets, and primary bile acids are converted to secondary bile acids by a part of gut bacteria (Wahlström et al. 2016). Bile metabolism can modulate gut microbiota maturation (van Best et al. 2020). Human oligosaccharides (HMOs) in maternal milk are among the most important factors to modulate gut bacteria (Lawson et al. 2020). A part of gut bacteria such as members of the genus *Bifidobacterium*, *Bacteroides* and *Akkermansia muciniphila* have specific enzymes to use HMO (Aakko et al. 2017; Kostopoulos et al. 2020). Conversely, diets and antibiotics also affect gut microbiota (Gentile and Weir 2018; Mu and Zhu 2019). Ingredients of diets such as fibre and fat or foods such as fermented milk can modulate gut microbiota (Daniel et al. 2014; Makki et al. 2018; Veiga et al. 2014). Diet can also alter human gut microbiota reproducibly (David et al. 2014).

Gut bacteria can strongly affect host systems involved in homeostasis such as metabolism and the central nervous and immune systems (Marchesi et al. 2016; Rutsch et al. 2020). Concerning the immune-metabolic axis, gut bacteria are associated with metabolic disorders such as obesity and type II diabetes (Dabke et al. 2019). In the brain–gut axis, multiple sclerosis and Alzheimer’s diseases are strongly correlated with gut microbiota (Chen et al. 2016; Vogt et al. 2017). Gut microbiota is also associated with blood glucose regulation via enteric neurons (Muller et al. 2020). From a comparison study of gut microbiota between healthy volunteers and patients, gut bacteria associated with host health such as *Akkermansia muciniphila* (improvement of metabolic disorder) have been reported (Depommier et al. 2019; Everard et al. 2013; Plovier et al. 2017). That is why gut microbiota is recognised as a superorgan in the human body (Putignani et al. 2014). It is of interest to understand how gut bacteria interact with human health and how the gut stimulates beneficial bacteria that inhabit a gut.

*A*. *muciniphila*, isolated from human faeces, can degrade mucin and use it as a sole carbon and nitrogen source (Derrien et al. 2004). The abundance of *A*. *muciniphila* is inversely associated with obesity, diabetes and inflammation (Hansen et al. 2012; Santacruz et al. 2010; Schneeberger et al. 2015). The reverse effect of *A*. *muciniphila* on obesity and diabetes is becoming clear by animal and human studies (Depommier et al. 2019; Plovier et al. 2017). *A*. *muciniphila* can also induce host adaptive immune response (Ansaldo et al. 2019). These studies make *A*. *muciniphila* a next-generation beneficial microbe. Hence, our health needs to stimulate *A*. *muciniphila* in the gut. This review gives an update on current knowledge of the stimulation factor of *A*. *muciniphila*. The effect of host-derived substance, bacteria and diets on the change in the gut microbial niche of *A*. *muciniphila* is reviewed.

## Interaction with host-derived substances

**Fig. 1:**
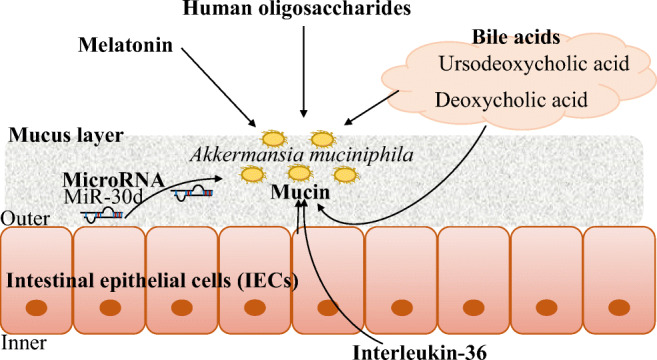
The host-derived substances modulate *A*. *muciniphila*

Carbon and nitrogen sources are the most important nutrients for microbes and those that reside in the gut environment. Figure 1 illustrates the important host-derived substances for *A. muciniphila*. Mucin is the most important substance for mucin-degrading bacterium *A*. *muciniphila* (Ottman et al. 2017). Administration of mucin stimulates *A*. *muciniphila*, so that *A*. *muciniphila* prefers mucin to other sugar such as glucose and mannose (Berry et al. 2015). Especially, *A*. *muciniphila* may prefer sulfated mucin because the higher percentage of sulfated mucin is positively associated with the abundance of *A*. *muciniphila* (Earley et al. 2019). On the mucus layer, oxygen is diffused from intestinal epithelial cells (IECs). *A*. *muciniphila* also adapts to oxic–anoxic interface using cytochrome system (Ouwerkerk et al. 2016). The interaction between *A*. *muciniphila* and mucin has been reviewed in more detail (Geerlings et al. 2018). Mucin is secreted by IECs. IECs are key cells of gut bacteria interaction because of their gut homeostasis functions such as mucosal barriers and immunological mediators (Wittkopf et al. 2014). There is a unique substance derived from IECs, which can stimulate the growth of *A*. *muciniphila*, ‘microRNA’.

IECs and other cells such as immune cells, adipocytes and other epithelial cells produce microRNAs. MicroRNAs are small non-coding RNAs, a class of endogenous RNAs with 21–25 nucleotides (O’Brien et al. 2018). MicroRNAs are observed in the faecal samples and body fluids such as serum, milk and saliva (Duran-Sanchon et al. 2020; Gallo et al. 2012; Kroh et al. 2010; Mirza et al. 2019). The host’s gene expression and the gut microbiota are regulated by microRNAs (Gennarino et al. 2009). The disturbance of gut microbiota composition is observed in IEC microRNA-deficient mice (Liu et al. 2016). MiR-515-5p and miR-1226-5p can affect the growth of *Fusobacterium nucleatum* and *Escherichia coli*, respectively. In the case of *A*. *muciniphila*, oral administration of miR-30d to mice affects this microorganism’s physiology by regulating the expression of gene encoding lactase of *A*. *muciniphila* leading to the bloom of *A*. *muciniphila* (Liu et al. 2019). Different microRNAs have a specific effect on each gut bacteria. Further study on the interaction between microRNA and gut bacterial species could lead to innovative techniques for modulating the gut microbiota’s targeted microorganisms.

Breast milk can modulate gut microbial composition at an early life stage (Moossavi et al. 2019). Here breast milk is defined as physiological substances because it contains sugar (oligosaccharides), proteins, vitamins, hormones, cytokines and even bacteria (Ballard and Morrow 2013; Moossavi et al. 2019). Concerning the effect of breast milk on gut bacteria, HMOs are well known to stimulate *Bifidobacterium* spp. (Lawson et al. 2020). Although the abundance of *Bifidobacterium* spp. in the breast-fed infants was higher than that in formula-fed infants, the lower abundance of *A*. *muciniphila* was observed in breast-fed infants (Bergström et al. 2014). Interestingly, *A*. *muciniphila* can use HMOs (Kostopoulos et al. 2020). Dual hydroxylation of glycan in mucin and breast milk may confer an ability for getting a niche in the gut mucosal environment to *A*. *muciniphila*. Goat milk also could increase the population of *A*. *muciniphila* in mice although the mechanism is unknown (Kao et al. 2020).

The comparison study on the interaction between metabolic disorder and microbiome newly demonstrates the effect of hormone and cytokine on the abundance of *A*. *muciniphila*. Melatonin, known as a sleep-promoting hormone secreted by the enigmatic pineal gland (Pandi-Perumal et al. 2005), prevents obesity via gut microbiota in high-fat-fed mice (Xu et al. 2017). This study also shows that melatonin treatment could increase the abundance of genus *Akkermansia*. The abundance of *A*. *muciniphila* is regulated by interleukin-36 (IL-36) cytokine (Giannoudaki et al. 2019). The knockout of IL-36 receptor antagonist, known to inhibit the effect of IL-36, can cause an increase in mucin production leading to the abundance of *A*. *muciniphila* and the reduction of weight gain and metabolic dysfunction in mice.

Bile metabolism is strongly associated with gut microbiota because primary bile acids secreted by the host are converted to secondary bile acids by gut bacteria-harbouring bile modification enzymes associated with bile hydroxylation and deconjugation (Wahlström et al. 2016). The growth of *A*. *muciniphila* is increased by a secondary bile acid (deoxycholic acid (DCA)) (Hagi et al. 2020). DCA also increases the expression of MUC2 in human colon carcinoma cells (Song et al. 2005). Another bile acid, ursodeoxycholic acid (UDCA), can increase the abundance of *A*. *muciniphila* in mice (Van den Bossche et al. 2017). Secondary bile acid may be a key bile acid for *A*. *muciniphila* to survive in the gut. A part of the bile acid-resistant mechanism in *A*. *muciniphila* has been clear (Hagi et al. 2020). Transcriptomic analysis showed that the treatment of ox-bile upregulated the expression of genes encoding HlyD (membrane fusion protein)-ABC and RND (resistance-nodulation-cell division protein family) type transporters. An ABC transporter inhibitor (orthovanadate) and RND-type transporter inhibitor (PaβN (Phe-Arg β-naphthylamide dihydrochloride)) could reduce the tolerance against bile acids. These transporters play an important role in the bile acid tolerance of *A*. *muciniphila*.

The suppressed gastric acid production by proton pump inhibitor (PPI) can alter gut bacterial composition in humans (Jackson et al. 2016). PPIs might weaken a barrier of gastric acid to protect bacterial invasion from the external environment. Interestingly, PPI use is also positively associated with the high-fat mass index and negative abundance of *A*. *muciniphila* (Davis et al. 2020). However, whether IPPs could affect the change in abundance of *A*. *muciniphila* directly is unclear. Another medicine, metformin used in the treatment of type 2 diabetes, affected the increased population and change in gene expression of *A*. *muciniphila* (Shin et al. 2014; Wu et al. 2017). The change in gut microbiota by metformin may be associated with host homeostasis because the level of bile acids in plasma and SCFAs in faeces could be increased by metformin. Medicines used for metabolic syndrome could affect *A*. *muciniphila* via a change in host metabolism such as bile acids although the detailed mechanisms are unknown. These results imply that the impact of transporter inhibitors on commensal gut microbiota including *A*. *muciniphila* needs to be investigated for our health.

## Interaction with bacteria

**Fig. 2:**
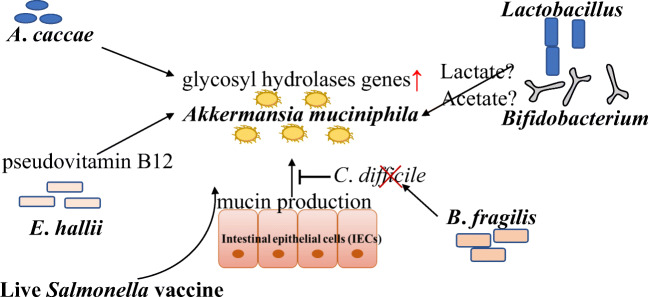
The interaction between *A*. *muciniphila* and other bacteria

Various gut bacteria such as *A*. *muciniphila* inhabit the mucus layer and interact with each other. Figure 2 illustrates the interplay between *A*. *muciniphila* and other bacteria. Some bacteria cannot degrade mucin, so non-mucin-degrading bacteria benefit from mucin-degrading bacteria. *A*. *muciniphila* produces sugars derived from mucus and SCFAs such as acetate and propionate. Non-mucus-degrading bacteria such as *Anaerostipes caccae*, *Eubacterium hallii* and *Faecalibacterium prausnitzii* use sugars degraded from mucin by *A*. *muciniphila* for their growth. The growth of *A*.*caccae* and butyrate production was supported by mucin degradation of *A*. *muciniphila* (Chia et al. 2018). Interestingly, *A*. *caccae* induces the increased expression of mucin degradation genes and reduced expression of ribosomal genes in *A*. *muciniphila*. In another bacterial interaction, 1,2-propanediol produced by *A*. *muciniphila* is used by *E*. *hallii* for the production of propionate. In return for sugars and SCFA, pseudovitamin B12 required for propionate metabolism is given by *E*. *hallii* (Belzer et al. 2017). *A*. *muciniphila* also has a cobamide remodelling enzyme CbiR (Mok et al. 2020). In the presence of a vitamin B12 analogue cobinamide, *A*. *muciniphila* can produce pseudovitamin B12 using CbiR. Another study shows that another *A*. *muciniphila* strain could produce vitamin B12 (ATTC BAA-835 strain cannot synthesise) although vitamin B12 from others promoted propionate production (Kirmiz et al. 2020). There are some vitamin B12 analogues in human faeces (Allen and Stabler 2008). *A*. *muciniphila* might use cobalamin analogues and affect vitamin B12 metabolism in the gut. Unique cobamide metabolism may be acquired to inhabit a complex ecological niche. These metabolic cross-feeding networks are important for regulating microbial composition and host health.

There are several papers on the effect of probiotic bacteria on the growth of *A*. *muciniphila*. The administration of mixture composed of *Lacticaseibacillus rhamnosus* LMG S-28148 and *Bifidobacterium animalis* subsp. *lactis* LMG P-28149 improved the abundance of *A*. *muciniphila* associated with decreased glucose and insulin levels in obese mice (Alard et al. 2016). Acetate and lactate derived from the probiotics might stimulate the growth of *A*. *muciniphila* because acetate and the acetate-conjugated portion of the drug could boost the growth of *A*. *muciniphila* (Daisley et al. 2020). In a human study, the administration of *L. rhamnosus* HN001 and *B*. *longum* BB536 increases the abundance of *A*. *muciniphila* (Toscano et al. 2017). Dizman et al. demonstrated that fermented milk with *B*. *animalis* could improve the abundance of *A*. *muciniphila* in metastatic renal cell carcinoma patients (Dizman et al. 2021). These species may interact with *A*. *muciniphila* although the interaction mechanism is unknown.

A dose of *B*. *fragilis* increased the abundance of *A*. *muciniphila* in a mouse model of *Clostridium difficile* infection (Deng et al. 2018). Cross-feeding interaction via SCFAs between *A*. *muciniphila* and *B*. *fragilis* or the inhibition of *C*. *difficile*-induced apoptosis and Muc2 and ZO-1 loss in colon cells may contribute to the increase in *A*. *muciniphila*. In chicken, live *Salmonella* vaccine increased a mucus level resulting in an abundance of *A*. *muciniphila* (Redweik et al. 2019). Change in host metabolism or production of SCFAs by probiotic or vaccine effects might improve the growth of *A*. *muciniphila* in the gut.

## Interaction with diets

**Fig. 3:**
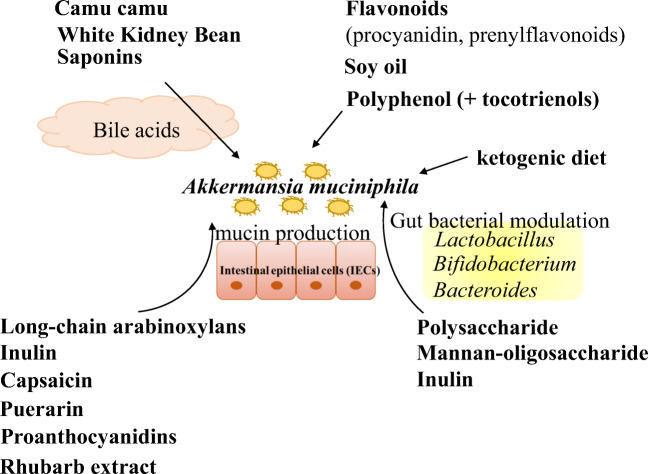
The boost of *A*. *muciniphila* by diets

*A*. *muciniphila* uses carbohydrates derived from mucin composed of fucose, galactose, N-acetylgalactosamine (GalNAc) and N-acetylglucosamine (GlcNAc) (Ottman et al. 2017). How can other carbohydrates help *A*. *muciniphila* growth? Xylo-oligosaccharide, fructo-oligosaccharides, arabinoxylan and inulin are known as prebiotics, resulting in improved beneficial bacterial growth such as bifidobacteria and lactobacilli (McLaughlin et al. 2015). Figure 3 describes the diets or their components associated with the boost of *A*. *muciniphila*. Long-chain arabinoxylans and inulin affect the higher production of mucin leading to an increased faecal abundance of *A*. *muciniphila* in humanised rats (Van den Abbeele et al. 2011). Induced-mucin production by these components is associated with the abundance of mucin-degrading *A*. *muciniphila* because *A*. *muciniphila* cannot grow on inulin. Mannan-oligosaccharide also can increase the abundance of *A*. *muciniphila* as well as that of *B*. *acidifaciens*, *L. gasseri* and *B*. *pseudolongum* in high-fat diet-fed mice, resulting in the attenuation of metabolic disorders (Wang et al. 2018). In addition to oligosaccharides from dietary fibres, the abundance of *A*. *muciniphila* increased by polysaccharide from seaweed (Enteromorpha Clathrata) as well as *Bifidobacterium* spp. and *Lactobacillus* spp. (Shang et al. 2018). Gut bacteria such as *Bifidobacterium* and *Bacteroides* can degrade natural polysaccharides (Flint et al. 2008). Although it is not clear if *A*. *muciniphila* can degrade polysaccharides, bacterial degradation of polysaccharides may support the growth of *A*. *muciniphila*. A low carbohydrate diet, ketogenic diet, alters gut microbial composition with increased *A*. *muciniphila* in mice (Ma et al. 2018). The change in the gut immune system might affect *A*. *muciniphila* because ketogenic diets also contribute to the decreased intestinal Th17 (Ang et al. 2020). The promoted mucin production by Th17 attenuation might affect the abundance of *A*. *muciniphila* because the correlation between an inhibited Th17 differentiation and an increased mucin production has been indicated (Cha et al. 2010).

Plants such as vegetables, beans and herbs have plenty of phytochemicals and the effect of plants or their extractions including phytochemicals on the host health and gut microbiota has been studied (Yin et al. 2019). The increase in *A*. *muciniphila* was observed in high-fat/high-sucrose (HFHS)-fed mice by the treatment of camu camu (*Myrciaria dubia*) which is a native fruit of the Amazon (Anhê et al. 2019). The treatment of camu camu affected the composition of bile acids and the increase in *A*. *muciniphila*, resulting in the prevention of obesity and metabolic syndrome. Interestingly, the abundance of *A*. *muciniphila* is positively correlated with the secondary bile acids such as Tauro-conjugated DCA and UDCA. The secondary bile acids such as DCA and lithocholic acid can activate TGR5. TGR5, highly expressed in the gallbladder, brown adipose tissue and other tissues, is a bile acid-responsive receptor involved in host metabolism. The interaction between *A*. *muciniphila*, bile metabolism and camu camu might be related to the anti-obesity effect. The increase in *A*. *muciniphila* and the change in bile metabolism were observed by the treatment of white kidney bean (*Phaseolus vulgaris L*.) (Neil et al. 2019). Saponins from *Agave salmiana* also modulate bile acids and cholesterol transport systems in the liver of mice and increased *A*. *muciniphila* (Leal-Díaz et al. 2016). As described above in this review, UDCA treatment can increase the abundance of *A*. *muciniphila* in mice (Van den Bossche et al. 2017). Our group showed that DCA increased the growth of *A*. *muciniphila* in vitro and its bile acid-resistant metabolism (Hagi et al. 2020). These studies indicate that the change in bile acids, especially UDCA, by the treatment of camu camu could increase the abundance of *A*. *muciniphila*, and the interaction between secondary bile acids and *A*. *muciniphila* might affect the host bile acid metabolism. The modulation of mucus layer by diets, such as capsaicin, puerarin in the root of *Pueraria lobate* and wild blueberry proanthocyanidins (Rodríguez-Daza et al. 2020; Shen et al. 2017; Wang et al. 2019), may cause the increase in *A*. *muciniphila*. The stimulation of IECs by rhubarb extract also might contribute to the increase in *A*. *muciniphila* (Neyrinck et al. 2017). In other reports, flavonoids from apple (procyanidin) and hops (prenylflavonoids) could stimulate the growth of *A*. *muciniphila* leading to the improvement of metabolic syndrome (Fukizawa et al. 2020; Hamm et al. 2019; Masumoto et al. 2016). Polyphenol derived from cranberry and green tea (synergistic effect with annatto-extracted tocotrienols) is also a key component in a diet that stimulates the growth of *A*. *muciniphila* (Anhê et al. 2015; Elmassry et al. 2020). Polyphenols such as catechin have antibacterial activities (Ikigai et al. 1993). The antibacterial activity might reshape gut microbiota, which is favourable for *A*. *muciniphila*. In addition to the phytochemicals described above, soy oil also could affect the abundance of *A*. *muciniphila* (Patrone et al. 2018).

## Conclusions

In conclusion, this article reviewed the effects of several factors such as host-derived substance, bacteria and diets on the growth of *A*. *muciniphila*. Mucin is a sole carbon and nitrogen source for *A*. *muciniphila*. Mucin production-stimulated factors, inulin and secondary bile acid may be related to the bloom of *A*. *muciniphila*. The growth of *A*. *muciniphila* also depends on a host-derived substance such as bile acids, microRNA and oligosaccharides. Gut bacteria and probiotic bacteria (especially bifidobactera and lactobacilli) also could affect the growth of *A*. *muciniphila*. To boost the growth of *A*. *muciniphila* in the gut, recent studies have investigated the effect of diet on its growth. Polysaccharides and inulin known as prebiotics could increase the abundance of *A*. *muciniphila*. This result may be associated with the change in gut environment by prebiotics resulting in the improvement of beneficial bacterial growth such as bifidobacteria and lactobacilli. In addition to bacterial interaction, bile acids are also considered one of the growth modulating factors of *A*. *muciniphila* because it is associated with gut bacterial composition, and secondary bile acid metabolism could be affected by diet camu camu. Altogether, these pieces of knowledge should help develop future treatment strategies to modulate health through increasing abundance and activity of *A*. *muciniphila* in the gut.
